# Single-center study of familial papillary thyroid cancer in China: surgical considerations

**DOI:** 10.1186/s12957-015-0519-4

**Published:** 2015-03-21

**Authors:** Shangtong Lei, Da Wang, Junna Ge, Hao Liu, Donghui Zhao, Guoxin Li, Zihai Ding

**Affiliations:** Department of General Surgery, Nanfang Hospital, No, 1838 North Guangzhou Avenue, Guangzhou, 510515 Guangdong China; Anatomical Institute of Minimally Invasive Surgery, Southern Medical University, No. 1838 North Guangzhou Avenue, Guangzhou, 510515 Guangdong China

**Keywords:** Familial papillary thyroid cancer, Aggressiveness, Clinicopathologic characteristics, Surgical considerations

## Abstract

**Background:**

Whether familial papillary thyroid cancer (FPTC) is more aggressive than sporadic counterpart remains elusive, and the optimal clinical approach for FPTC is yet to be established. In this study, we investigated familial occurrence of PTC in China and reviewed our experience of its surgical treatment.

**Methods:**

The clinical records of 248 consecutive patients with an established diagnosis of PTC who were admitted to Nanfang Hospital for thyroidectomy between January 2011 and June 2013 were analyzed in this study. Patients included 66 males and 182 females, aged 11 to 76 years.

**Results:**

Twenty-two patients (8.9%) with a positive family history were confirmed. Patients with FPTC had a predilection for female subjects and tended to be younger than other patients, but the difference was not significant (*P* = 0.0514 and *P* = 0.168). They were more likely to present large tumors (*P* = 0.0024), multifocality (familial vs. sporadic: 54.50% vs. 26.50%; *P* < 0.006), local invasion (81.8% vs. 23.9%; *P* < 0.001), and malignant lymph nodes (63.6% vs. 33.6%; *P* = 0.005). Univariate and multivariate analyses identified that a positive family history was an independent risk factor for local invasion (OR: 5.683; 95% CI: 2.056 to 15.707; *P* = 0.001), malignant lymph nodes (OR: 3.005; 95% CI: 1.046 to 8.630; *P* = 0.041) in FPTC patients. Kaplan-Meier survival curves revealed that an aggressive surgical strategy was associated with a better relapse-free survival than conventional one (*P* = 0.032).

**Conclusions:**

FPTC is more likely to possess aggressive features than sporadic counterparts. Thus, screening of at-risk families is essential to aid in earlier recognition. An aggressive surgical strategy appeared to be the more effective therapy. However, sufficient detailed interrogation and long-term follow-up of the patients and their family are necessary for providing individualized recommendations for clinical management.

## Background

Thyroid cancer is the tenth most common cancer in China, accounting for approximately 2.29% of all cancers according to the Chinese Cancer Registry Annual Report 2012 [1]. Over 95% of all thyroid cancers are non-medullary thyroid cancers (NMTCs) that arise from thyroid follicular cells, which consist of four histological types: papillary (85%), follicular (11%), Hurthle cell (3%), and anaplastic (1%) [2]. Little evidence has been known about the epidemiology of rare histological types. The incidence of papillary thyroid cancer (PTC) is high and has been continually increasing in the past decades in the countries from East Asia and the West. Though most PTC arises sporadically, it is estimated that a familial origin is present in 3.5% to 6.2% of cases, and the rate in women is twice that in men [3]. There are some established risk factors, such as ionizing radiation, iodine deficiency, and goiter. In addition, a number of studies have provided strong evidence for a genetic basis of PTC ever since the first description of familial papillary thyroid cancer in 1955 by Robinson and Orr [4].

Familial papillary thyroid cancer (FPTC) is now defined by a diagnosis of at least two first-degree relatives with PTC without another familial syndrome. This excludes PTC of distinct types that can be a component in some Mendelian-inherited cancer syndromes, such as familial adenomatous polyposis, Cowden’s syndrome, Werner syndrome, Carney complex, and Peutz-Jeghers syndrome. Cases of PTC are documented to be associated with a younger age at presentation; they are more aggressive than sporadic PTC and associated with higher recurrence [2]. However, registry-based data are rare.

There is no general agreement on the best surgical management strategy for FPTC. Therefore, the aim of the study is to investigate the occurrence of FPTC in a single center in China and identify factors in our database that were taken into consideration for surgical treatment of FPTC. The results may contribute to establishment of an optimal clinical approach for patients with this type of cancer.

## Methods

### Patients

A total of 248 consecutive patients admitted to Nanfang Hospital with an established diagnosis of PTC to undergo thyroidectomy between January 2011 and June 2013 were included in this study. All study patients were questioned regarding the presence or absence of similarly affected family members. A family history was considered positive when patients had at least two first-degree relatives with PTC. Family history and medical information, including demographics, radiation exposure, additional syndromes, surgical information, and pathological diagnosis, were recorded in the database. Stage of thyroid cancer was described according to the 2010 tumor node metastasis (TNM) classification of malignant tumors by the American Joint Committee on Cancer (AJCC). All patients underwent total thyroidectomy and modified neck dissection as the American Thyroid Association recommended except 13 FPTC cases after January 2012. These 13 patients were categorized into the aggressive surgery group (AG) and the remaining FPTC patients into the conventional surgery group (CG). An aggressive surgical strategy was used in AG; briefly, we performed a prophylactic central neck dissection in any FPTC patient with a primary tumor of >1 cm in size or smaller tumor but exhibiting local invasion including minimal extrathyroidal extension or invasion to adjacent organs and a lateral neck dissection should be considered if enlarged and suspicious nodes were detected in the cervicolateral compartment in advanced carcinomas. Complete follow-up, ranging from 16 to 36 months, was available for all FPTC patients.

### Ethics statement

All patients provided written informed consents for the storage and use of their data. This study was approved by the Ethics Committee of Nanfang Hospital, Southern Medical University, China.

### Statistical analysis

Statistical analysis was performed using the SPSS statistical software package (SPSS® version 16.0) (SPSS Inc.; Chicago, IL, USA). Where appropriate, the Mann–Whitney *U*-test, two-tailed *t*-test, and Fisher’s exact test was used for comparisons between two independent groups. A logistic regression model was used to assess odds ratios (ORs) and identify factors that familial risks independently predict. A difference between two mean values was considered significant when *P* < 0.05.

## Results

A total of 248 consecutive patients were entered in the study, including 66 males and 182 females of aged 11 to 76 years (mean age: 40.40 years) (Table 1). Among them, nodular goiter occurred in 106 cases, thyroid adenoma in 86, and Hashimoto’s thyroiditis in 38. Median number of total removed lymph nodes was 9. And the numbers of patients with stage I (<45), II (<45), I (≥45), II (≥45), III (≥45), and IV (≥45) thyroid cancer were 146, 8, 28, 10, 26, and 30, respectively, according to AJCC classification. The familial relationship between the affected family members was illustrated in Table 2.

**Table 1:** **Baseline characteristics of patients included in this study**

**Characteristics**	**Patient data (** ***n*** **= 248)**
Gender	
Male	66
Female	182
Age (years, mean ± SD)	40.40 ± 13.35
<45	158
≥45	94
Histological types: papillary	248
Combined with benign thyroid diseases:	
Nodular goiter	106
Thyroid adenoma	86
Hashimoto’s thyroiditis	38
Median number of tLNs (range)	9(0 to 119)
Median number of mLNs (range)	0(0 to 33)
AJCC stage	
<45 I:II	146:8
≥45 I:II:III:IV	28:10:26:30

SD: standard deviation, tLN: total resected lymph nodes, mLN: metastatic lymph nodes, AJCC: American Joint Committee on Cancer.

**Table 2:** **Comparison of parent–child relationship in FPTC patients**

**Parameter**	**Gender(male/female)**	**Age at diagnosis (years, mean ± SD)**	**Tumor size (cm, mean ± SD)**	**Multifocality**	**Rapid proliferation**	**Local invasion**	**Lymph node affection**
First generation (*n* = 12)	0/12	35.17 ± 12.90	2.10 ± 1.35	8(66.7%)	0(0.00%)	12(100.0%)	10(83.3%)
Second generation (*n* = 10)	2/8	38.40 ± 14.93	2.40 ± 1.80	4(40.0%)	4(40.0%)	6(60.0%)	4(40.0%)
*P*	0.195	0.592	0.670	0.391	0.029	0.029	0.074

Of the 248 study patients, 22 were confirmed with a positive family history. Comparing clinicopathologic characteristics between familial and sporadic PTC patients in the present study, we found that patients with any positive family history were more likely to exhibit tumors of larger size (36.4% vs. 12.4%; *P* = 0.0024), multifocality (54.50% vs. 26.50%; *P* = 0.006), local invasion (81.8% vs. 23.9%; *P* < 0.001), and malignant lymph nodes (63.6% vs. 33.33%; *P* = 0.005) (Table 3). However, familial occurrence of PTC was not significantly associated with age at diagnosis, gender, median number of total resected lymph nodes, median number of metastatic lymph nodes, combination with benign thyroid diseases, or AJCC stage.

**Table 3:** **Clinicopathologic characteristics of familial and sporadic PTC patients**

**Characteristics**	**Familial (** ***n*** **= 22)**	**Sporadic (** ***n*** **= 226)**	***P*** **value**
Gender			0.0514
Male	2(9.10%)	64(28.3%)	
Female	20(91.0%)	162(71.7%)	
Age (years, mean ± SD)	36.64 ± 13.95	40.77 ± 13.30	0.168
Tumor size			*0.0024*
>4 cm	8(36.4%)	28(12.4%)	
≤4 cm	14(63.6%)	198(87.6%)	
Tumor aggressiveness: *n*(%)			
Multifocality	12(54.50%)	60(26.50%)	*0.006*
Rapid proliferation	4(18.2%)	32(14.0%)	0.597
Local invasion^a^	18(81.8%)	54(23.9%)	*<0.001*
Malignant lymph nodes	14(63.6%)	76(33.6%)	*0.005*
Number of tLNs: median (range)	7(0 to 37)	9(0 to 119)	0.660
Number of mLNs: median (range)	1(0 to 19)	0(0 to 33)	0.125
Combined with benign thyroid diseases	14(63.6%)	136(60.2%)	0.751
AJCC stage			
Age <45			1.00
I	12(54.5%)	134(59.3%)	
II	0	8(3.54%)	
Age ≥45			0.306
I + II	6(27.3%)	32(14.2%)	
III + IV	4(18.2%)	52(23.0%)	
Relapse	3(13.6%)	14(6.2%)	0.182

SD: standard deviation, tLN: total resected lymph nodes, mLN: metastatic lymph nodes, AJCC: American Joint Committee on Cancer. ^a^Local invasion including minimal extrathyroidal extension and invasion to esophagus, larynx, recurrent laryngeal nerve, and subcutaneous soft tissues.

To further validate the effect of familial occurrence of PTC on tumor aggressiveness, univariate and multivariate analyses identified that a positive family history was an independent risk factor for local invasion (OR: 5.683; 95% CI: 2.056 to 15.707; *P* = 0.001) (Table 4) and malignant lymph nodes (OR: 3.005; 95% CI: 1.046 to 8.630; *P* = 0.041) (Table 5) in FPTC patients but not for tumor size (Table 6) and multifocality (Table 7).

**Table 4:** **Univariate and multivariate analyses of risk factors predicting local invasion in FPTC patients**

	**Univariate** ^**a**^			**Multivariate** ^**b**^		
	**OR**	**95% CI**	***P***	**OR**	**95% CI**	***P***
Group	8.494	3.166, 22.786	*<0.001*	5.683	2.056, 15.707	*0.001*
Gender	2.105	1.046, 4.234	*0.037*	2.010	0.957, 4.223	0.065
Age	1.857	1.058, 3.259	*0.031*	2.306	1.218, 4.365	*0.010*
Tumor size	1.975	0.935, 4.172	*0.075*	1.425	0.617, 3.290	0.407
Multifocality	1.024	0.555, 1.890	0.940			
Rapid proliferation	1.991	0.786, 5.042	0.146			
Metastatic lymph nodes	2.431	1.379, 4.288	*0.002*	2.542	1.345, 4.804	*0.004*

Group: familial vs. sporadic, Gender: female vs. male, Age (years): ≥45 vs. <45, Tumor size (cm): >4 vs. ≤4, Multifocality: yes vs. no, Rapid proliferation: yes vs. no, Metastatic lymph nodes: yes vs. no; OR: odds ratio, CI: confidence interval. ^a^Hazard ratios in univariate models; ^b^Hazard ratios in multivariable models.

**Table 5:** **Univariate and multivariate analyses of risk factors predicting malignant lymph nodes in FPTC patients**

	**Univariate** ^**a**^			**Multivariate** ^**b**^		
	**OR**	**95% CI**	***P***	**OR**	**95% CI**	***P***
Group	3.454	1.388, 8.593	*0.008*	3.005	1.046, 8.630	*0.041*
Gender	0.996	0.554, 1.788	0.988			
Age	0.457	0.260, 0.803	*0.006*	0.353	0.190,0.655	*0.001*
Tumor size	1.271	0.608, 2.659	0.524			
Multifocality	0.885	0.496, 1.581	0.681			
Rapid proliferation	0.340	0.162, 0.713	*0.004*	0.262	0.119, 0.575	*0.001*
Local invasion	2.431	1.379, 4.288	*0.002*	2.833	1.486, 5.399	*0.002*

Group: familial vs. sporadic, Gender: female vs. male, Age (years): ≥45 vs. <45, (cm): >4 vs. ≤4, Multifocality: yes vs. no, Rapid proliferation: yes vs. no, Local invasion: yes vs. no; OR: odds ratio, CI: confidence interval. ^a^Hazard ratios in univariate models; ^b^Hazard ratios in multivariable models.

**Table 6:** **Univariate and multivariate analyses of risk factors predicting larger tumor size in FPTC patients**

	**Univariate** ^**a**^			**Multivariate** ^**b**^		
	**OR**	**95% CI**	***P***	**OR**	**95% CI**	***P***
Group	2.652	0.958, 7.341	*0.061*	2.122	0.726, 6.204	0.169
Gender	1.208	0.518, 2.821	0.662			
Age	2.043	0.958, 4.236	*0.055*	1.927	0.920, 4.036	0.082
Multifocality	1.467	0.683, 3.154	0.326			
Rapid proliferation	1.223	0.402, 3.719	0.723			
Local invasion	1.975	0.935, 4.172	*0.075*	1.564	0.707, 3.460	0.270
Metastatic lymph nodes	1.271	0.608, 2.659	0.524			

Group: familial vs. sporadic, Gender: female vs. male, Age (years): ≥45 vs. <45, Multifocality: yes vs. no, Rapid proliferation: yes vs. no, Local invasion: yes vs. no, Metastatic lymph nodes: yes vs. no; OR: odds ratio, CI: confidence interval. ^a^Hazard ratios in univariate models; ^b^Hazard ratios in multivariable models.

**Table 7:** **Univariate and multivariate analyses of risk factors predicting multifocality in FPTC patients**

	**Univariate** ^**a**^			**Multivariate** ^**b**^		
	**OR**	**95% CI**	***P***	**OR**	**95% CI**	***P***
Group	*2.306*	*0.947, 5.612*	*0.066*	2.306	0.947, 5.612	0.066
Gender	1.318	0.691, 2.517	0.402			
Age	1.576	0.898, 2.765	0.113			
Tumor size	1.467	0.683, 3.154	0.326			
Rapid proliferation	1.326	0.569, 3.088	0.514			
Local invasion	1.024	0.555, 1.890	0.940			
Metastatic lymph nodes	0.885	0.496, 1.581	0.681			

Group: familial vs. sporadic, Gender: female vs. male, Age (years): ≥45 vs. <45, Tumor size (cm): >4 vs. ≤4, Rapid proliferation: yes vs. no, Local invasion: yes vs. no, Metastatic lymph nodes: yes vs. no; OR: odds ratio, CI: confidence interval. ^a^Hazard ratios in univariate models; ^b^Hazard ratios in multivariable models.

**Figure 1:**
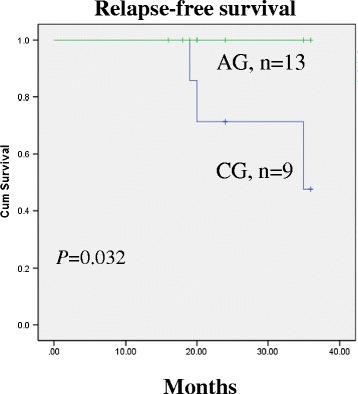
**Kaplan-Meier survival curves in FPTC patients grouped according to operation strategy.** A better relapse-free survival was observed in FPTC patients in AG than CG (*P* = 0.032). AG: aggressive surgery group, CG: conventional surgery group.

Because of the high rate of recurrence and lymphatic metastases seen in FPTC patients in the early period of this study and the association between reported appreciable death rate and recurrence, a more aggressive surgical strategy has been applied to treat FPTC since January 2012. Kaplan-Meier survival curves revealed that a more aggressive surgical strategy presented with a better relapse-free survival than conventional one (*P* = 0.032) (Figure 1).

## Discussion

Although frequently reported, whether FPTC patients shows a more aggressive behavior than their sporadic counterparts and whether FPTC requires more extended surgical procedures, such as total thyroidectomy and prophylactic neck dissection, remain inconclusive. Furthermore, there is no general recommendation to what extent non-affected family members should be screened for occult thyroid malignancy.

The prevalence of FPTC is difficult to establish due to the low incidence. We observed a familial incidence of 8.9% in 248 PTC cases enrolled in our study, and the age of FPTC patients was younger; whereas no significant association with age at diagnosis was noted [5]. In addition, the female:male ratio of FPTC patients was 5:1 in our study, higher than the ratio of 2:1 and 3:1 previously reported [6], whereas consistent with the findings from Orsenigo’s study [7]. It probably results from the sampling pool in this study.

FPTC is reported to behave more aggressively than sporadic pattern and correlate with early age of onset, increased incidence of benign thyroid nodules, multifocality, nodal involvement, and recurrence [8-10]. Mazeh et al. [10] found that compared with non-FPTC counterparts, patients with FPTC had similar tumor size but were significantly younger and more likely to present with multicentricity, malignant lymph nodes, and local invasion into surrounding tissues and higher recurrence rate. In a Japanese study, Ito [11] also regarded the occurrence of multicentricity and dramatically increased recurrence of thyroid cancer to be significant in their FPTC patients. However, lymph node metastasis, local invasion to surrounding tissues, and disease-free survival rate did not significantly differ between patients with sporadic PTC and FPTC [12].

Several characteristics in FPTC patients are associated with a poor prognosis. The primary feature is the aggressiveness of PTC. FPTC is associated with a higher risk of invasiveness and lymph node invasion. The recurrence of PTC is associated with a worse prognosis, even in a low-risk patient. The recurrence rate of FPTC is higher than that of sporadic PTC. The mortality of patients with recurrent PTC is up to 33.33% [2,9].

Contradicting results have been obtained in terms of familial and sporadic PTC. McDonald [5] statistically compared the clinical and pathologic data between FPTC and sporadic PTC patients using chi-square test and demonstrated no statistically significant trends in surgical pathological parameters, age at presentation, length of follow-up, or gender distribution. In agreement with Mazeh et al. [10], significant trends toward higher rates of repeated surgery and/or requiring additional radioactive iodine therapy, distant metastases, and deaths were seen in the familial PTC group. These aggressive features were the most apparent in certain families with three or more affected members [5].

Our findings, in agreement with several reports [2,8,9], indicated that FPTC is more aggressive than sporadic PTC, as determined by larger tumor size, increased local invasion, and malignant lymph node involvement. The presence of a positive family history is a significant risk factor of local invasion and lymph node metastases, to some extent, namely aggressiveness.

It is believed to have a genetic predisposition [13]. Linkage studies have identified the loci within specific families, including MNG1, TCO1, fPTC/PRN, and so on, whereas none of these candidates appears to account for a significant number of cases [14]. Triponez [15] demonstrated that patients who were diagnosed after an identified familial link has tended to yield better outcomes, highlighting the importance of early diagnosis and treatment for optimal outcomes. Until reliable genetic testing becomes available, obtaining a detailed family history is of great importance for recognition of any familial link to FPTC. Recently, Khara et al. reported a 3-year-old boy with familial PTC [16]. Since the patients in the second generation are frequently younger than the first generation counterparts, preoperative screening should be conducted at the age of 18 years according to expert consensus. Therefore, we suggest that all patients diagnosed with PTC should undergo a comprehensive history to identify potential familial forms of PTC.

To date, no clinical trials have been performed to establish a primary management strategy for FPTC. Although it remains controversial, many experts recommend the application of prophylactic thyroidectomy in patients at risk of FPTC [2,17]. Whether thyroidectomy should be performed mainly relies upon the information gained from a careful screening process of at-risk individuals and examining the course of disease within a family. Based on our experience, even lesions as small as ≤1 cm in size in FPTC patients can be associated with multifocality, invasion, and lymph node metastasis. We recommend that all patients with FPTC confirmed by biopsy should undergo total thyroidectomy regardless of tumor size. Our routine surgical procedure is first to excise the affected side, followed by excision of the contralateral side. Because of the high rate of nodal involvement in patients with FPTC, we recommend performing prophylactic central neck dissection in any patient with a primary tumor of >1 cm in size or smaller tumor but with local invasion to surrounding tissues. In advanced carcinomas, if preoperative ultrasound shows enlarged and suspicious nodes in the cervicolateral compartment, a lateral neck dissection should be considered. The high recurrence rate seen in these patients and the reported appreciable death rate associated with recurrence lead us to believe that almost all FPTC patients should be treated with postoperative radioactive iodine ablation, followed by thyroid hormone suppression. And shorter distance between the nodule and capsule has a greater risk of cervical lymph node metastasis, which is consistent with previous report [18].

Moreover, given genetic predisposition for thyroid cancer in these patients, any residual thyroid tissue is at risk of developing into a malignancy. Therefore, a more aggressive surgical strategy is recommended to obtain a better relapse-free survival in this study.

Besides genetic factors, environmental hazards should also be considered. Because it is elusive whether the incidence of malignancy is caused solely by genetic inheritance or by a combination of genetic predisposition and environmental influence, such as exposure to a low dosage of radiation, should not preclude the diagnosis of this disease [19,20].

However, sufficient detailed interrogation and long-term follow-up of the patients and their family are required to offer individualized recommendations according to the risk of PCT in each subject.

## Conclusions

FPTC is more likely to possess aggressive features than sporadic counterparts. Thus, screening of at-risk families is essential to aid in earlier recognition. An aggressive surgical strategy appeared to be the more effective therapy. However, sufficient detailed interrogation and long-term follow-up of the patients and their family are necessary for providing individualized recommendations for clinical management.
